# Supplementary material for: Metabolomic and transcriptomic signatures of prenatal excessive methionine support nature rather than nurture in schizophrenia pathogenesis
Source: Commun Biol. 2020 Jul 30;3:409. doi: 10.1038/s42003-020-01124-8 (PMC7393105; doi:10.1038/s42003-020-01124-8)
Supplement: Supplementary file 1 — Supplementary Information [file 42003_2020_1124_MOESM1_ESM.pdf]

Supplementary Figure 1a

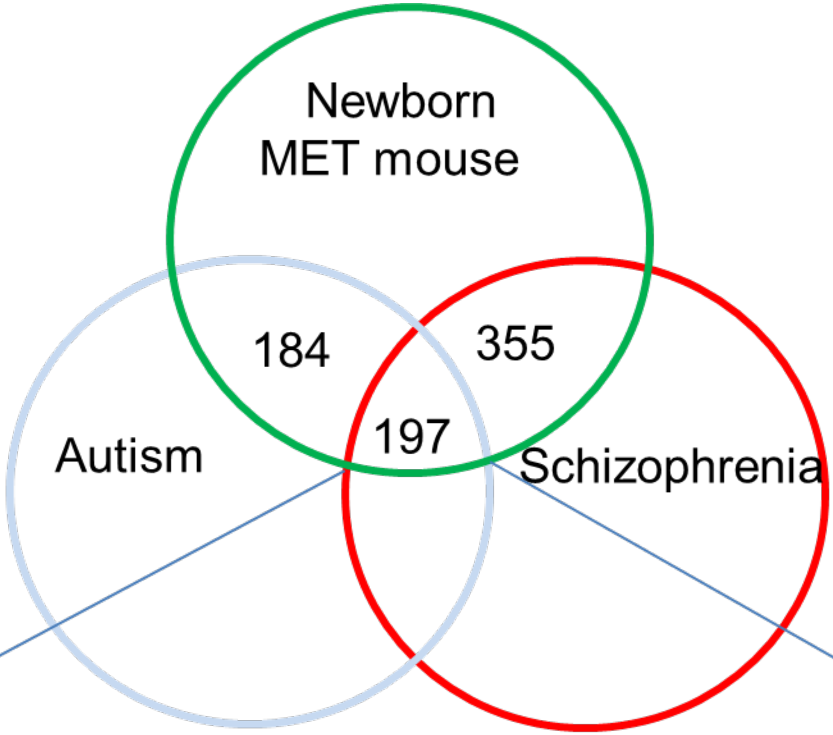

Gene_Symbol	MET.log2FC	MET.P-val	ASD.log2FC	ASD.p.value	SCZ.log2FC	SCZ.p.value
SERPINA3N	0.871843649	0.0002	1.1546822	0.0041541	0.9327256	1.14E-13
TPD52L1	- 0.731183242	5.42E-05	0.2569486	0.0048815	0.1262865	8.91E-06
SESN2	0.687060688	0.0002	0.2334936	0.0023911	0.1124706	2.01E-06
GFAP	0.650764559	0.0002	0.3272682	0.0034481	0.1417304	9.47E-05
NHP2L1	- 0.584962501	0.0107	-0.089384	0.0049057	-0.020432	0.0438139
HSPA5	0.565597176	9.20E-08	0.1809459	0.0295751	0.0590221	0.0206079
FDPS	0.475084883	0.0007	-0.123438	0.0011553	-0.036591	0.0023864
HSP90B1	0.464668267	1.90E-05	0.1390337	0.0082098	0.0473852	0.003538
MAP3K5	0.464668267	0.0105	0.1092901	0.0073146	0.0380869	0.0040021
GPR137B	0.432959407	0.0008	0.2148763	0.0003337	0.0451982	0.0191243

Supplementary Figure 1b

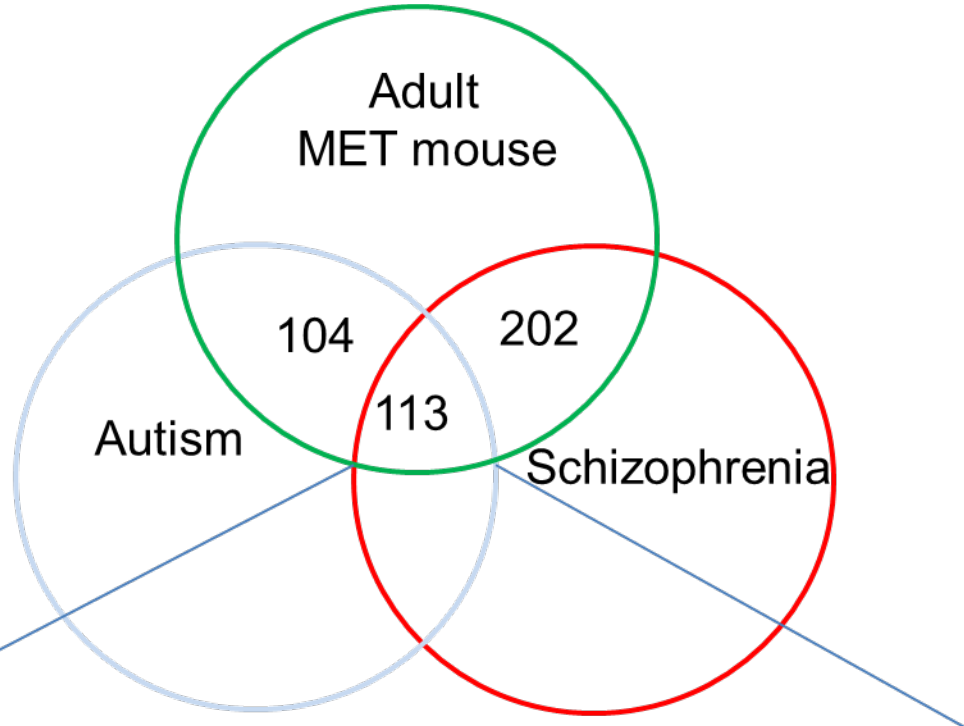

Gene_Symbol	MET.log2FC	MET.p.val	ASD.log2FC	ASD.p.value	SCZ.log2FC	SCZ.p.value
**TSNAX**	- 0.367371	0.03923	-0.17647	4.37E-05	-0.0349	0.009231
**SLC11A1**	0.31034	0.008124	0.473048	0.041415	0.354799	6.78E-07
**MC4R**	- 0.275007	0.009921	0.398237	0.000727	0.244528	4.09E-10
**RPL13A**	0.275007	0.006816	0.11091	0.007259	0.030688	0.021933
**MID1**	0.263034	0.030505	0.276535	0.000319	0.11375	3.26E-06
**PLSCR4**	0.263034	0.018974	0.312723	0.000991	0.161755	1.68E-07
**LDLRAP1**	0.250962	0.018588	0.20735	0.03451	0.10872	0.000861
**NXPH3**	0.238787	0.014262	-0.2556	0.000266	-0.09609	3.24E-05
**PPP1R14C**	0.238787	0.021609	-0.16866	0.001735	-0.03953	0.022549
**NBEAL2**	0.238787	0.043181	0.167713	0.003826	0.047478	0.011623

Supplementary Figure 1

Integrated mouse-human transcriptomic analysis reveals overlap between genes changed in MET mice and schizophrenia, and autism patients

a. Genes whose expressions are changed in the brains of newborn MET mice, schizophrenia patients, and autism patients

b. Genes whose expressions are changed in the brains of adult MET mice, schizophrenia patients, and autism patients

Supplementary Table 1. Newborn metabolite differential expressions

Metabolite name	pVal	Fold_Change
Malonyl-carnitine	1.42E-08	17.37784019
PC(34:3)	0.003225576	3.574221846
LPC(20:4)	0.009898235	3.175387368
LPC(22:6)	0.005861152	2.786656371
N-Acetylalanine	0.020758666	2.545295921
Ceramide (d34:0)	1.34E-05	2.509074836
LPC(18:3)	0.00156866	2.496785714
FA (16:1) (palmitoleic acid)	0.000190152	2.410129596
PC(p-36:4)/PC(o-36:5)	0.028178573	2.3496485
LPC (16:1)	0.005343272	2.20554044
LPC(16:1)	0.003248755	2.086582552
LPC(20:3)	0.000388322	2.059253928
FA (18:1) (oleic acid)	9.01E-05	2.051125414
LPE (20:4)	0.008111778	2.050065445
FA (22:6) (docosahexaenoic acid)	0.000259012	2.02644805
2-Propanamidoacetic acid	0.046561051	1.999669893
LPE(20:4)	0.00354443	1.990588567
LPE (22:6)	0.009771699	1.984216306
FA (20:4) (arachidonic acid)	0.000381253	1.926877101
Ergothioneine	0.017048832	1.904308617
FA (18:2) (linoleic acid)	0.00013315	1.900305243
FA (20:3) (homo-gamma-linolenic acid)	2.51E-05	1.887569714
FA (20:1) (eicosenoic acid)	6.35E-05	1.852139775
Cer(d42:1)	7.73E-06	1.845950745
GlcCer (d42:2)	5.01E-06	1.819345942
1-monopalmitin (palmitoyl glycerol)	0.014562486	1.800112296
FA (20:2) (eicosadienoic acid)	4.03E-05	1.774147029
LPC(18:1)	0.006345602	1.738119667
FA (20:5) (eicosapentaenoic acid)	0.00106048	1.731969101
FA (18:3) (linolenic acid)	0.000671825	1.724266992
Methylglycinate	0.047780322	1.714285714
LPC (18:2)	0.010863895	1.709048664
Isoleucine	0.02243996	1.701696475
glutathione	0.036729509	1.686834482
FA (20:3) (eicosatrienoic acid)	0.003619176	1.677735528
Ceramide (d40:2)	0.004406923	1.669829889
LPE (18:2)	0.002817457	1.668078859
PC(42:10)	0.035581665	1.658477842
Pro-Gln	0.048487458	1.646858033
Ceramide (d42:1)	1.99E-05	1.641631442
LPC (18:1)	0.022383612	1.638744964
LPC(14:0)	0.000919332	1.636602502
phosphoenolpyruvate	0.011634947	1.627368774
ceramide(d34:1)	0.000338275	1.625415475
GlcCer(d34:1)	0.018851089	1.625289291
ceramide(d40:1)	0.000218007	1.622755115

gamma-Glutamyl-L-glutamic acid	0.00521573	1.606305605
Ceramide (d40:1)	0.001221676	1.600645327
LPC (14:0)	0.010213989	1.59375
Ceramide (d41:1)	0.000375285	1.593629132
PC(37:4)	0.044537526	1.58276117
N,N,N trimethyllysine	0.00528629	1.580642806
Cytidine 5'-diphosphocholine	0.039031493	1.578072208
(2R)-3-Hydroxyisovaleroylcarnitine	0.02189903	1.57180386
FA (22:2) (docosadienoic acid)	1.67E-05	1.548834558
Ceramide (d33:1)	0.000788701	1.542713184
Ceramide (d34:1)	0.00020458	1.513003873
Citrulline	0.002934252	1.508178487
LPC(18:2)	0.005980702	1.505751451
Phenylalanine	0.046352501	1.494811879
LPE(18:0)	0.000155769	1.48537659
PG 34:2; PG 16:1-18:1	0.000740313	1.482888087
Serine	0.007874799	1.47509761
Proline	0.021629014	1.460093724
GlcCer (d38:1)	0.001267722	1.45780631
Ceramide (d38:1)	0.02988303	1.450890935
Ceramide (d42:2)	0.000204266	1.448209973
glycine	0.00249354	1.442933498
ceramide(d42:2)	0.007346085	1.439903868
Betaine	0.004378113	1.422000033
ceramide(d38:1)	0.019447342	1.419681051
FA (14:1) (physeteric acid)	0.000255794	1.419641477
LPC (16:0)	0.006337196	1.419327287
Guanosine 5'-tetraphosphate	0.01878964	1.381682644
LPC(16:0)	0.01926954	1.380779968
Threonine	0.021805471	1.378233512
S-Adenosyl-L-methionine	0.03780681	1.375611524
FA (14:0) (myristic acid)	0.000534169	1.352435553
Uridine 5'-monophosphate	0.038719373	1.338242444
LPE (16:0)	0.008000173	1.336140926
.beta.-Nicotinamide adenine dinucleotide (N	0.027644083	1.330447477
N-Acetylaspartylglutamic acid	0.031678685	1.318502034
Guanine	0.011415469	1.313997076
LPC(18:0)	0.041845937	1.300439441
PC (35:1)	0.001673869	1.299338698
Uridine	0.015460318	1.294951434
Ceramide (d34:2)	0.032542936	1.283715433
PC (36:5)	0.029199708	1.2761079
PC (40:4)	0.010632461	1.269081689
hippuric acid	0.037727235	1.265170038
LPC (18:0)	0.036701241	1.26043385
PC (40:8)	0.028178073	1.248804114
Aspartic acid	0.01055777	1.236658933

Ceramide (d32:1)	0.017833902	1.233891688
PC (34:4)	0.010881093	1.231711656
PC (35:4)	0.019959144	1.202626178
PC (34:3)	0.022476296	1.202529123
PC (38:3)	0.004024444	1.201970443
PC (36:1)	0.050758861	1.197213048
galacturonic acid	0.016558363	1.196483891
Inosine	0.023737185	1.177160032
PC (40:6) B	0.042754477	1.176184338
Adenine	0.02808395	1.17057998
PC (33:1)	0.033295672	1.167377348
PC (35:2)	0.027614	1.166033734
Creatine	0.022912855	1.131943573
stearic acid	0.042024025	-1.170770716
heptadecanoic acid	0.042367286	-1.190333949
4-hydroxybutyric acid	0.021800309	-1.190575734
3-deoxyhexitol NIST	0.01532955	-1.192243767
glutamic acid	0.005577824	-1.195742327
isocitric acid	0.049496068	-1.234030309
palmitic acid	0.016621365	-1.241368058
phosphoethanolamine	0.0340957	-1.250177482
orotic acid	0.017171631	-1.253148926
uracil	0.017249046	-1.297383776
fumaric acid	0.001518153	-1.320722296
alpha-aminoadipic acid	0.02215198	-1.323910483
leucine	0.011370697	-1.3252108
ribonic acid	0.002726209	-1.339101338
fucose	9.31E-06	-1.365242215
nicotinamide	0.001935704	-1.372416175
tyrosine	0.005086967	-1.385195884
adipic acid	0.008074106	-1.414739759
urea	0.002817654	-1.445821026
glycolic acid	0.022126295	-1.463490404
creatinine	0.012422587	-1.479562002
lactic acid	0.000265193	-1.525590211
SM(d36:1)	0.042836939	-1.564856564
tartaric acid	0.045332384	-1.574576271
tryptophan	0.03897947	-1.588580644
methionine sulfoxide	0.019447555	-1.596146166
DG(34:1)	0.049989843	-1.614326212
SM (d36:1)	0.004569146	-1.620467284
acetophenone	0.004900414	-1.658038825
Trigonelline	0.013151153	-1.66828188
glutamine	0.01743561	-1.9189826
hypoxanthine	0.001375521	-2.009659686
PG 44:12; PG 22:6-22:6;	0.018960217	-2.01024742
PC(p-38:4)/PC(o-38:5) B	0.006574967	-2.069430315

guanosine	0.000258347	-2.104031857
adenosine	0.030525858	-2.143022181
threose	0.000371668	-2.208040307
SM (d36:2)	0.002192205	-2.340853284
trans-4-hydroxyproline	0.036131287	-3.781165819
Sucrose	0.018709327	-4.654283145

Supplementary Table 2. Metaboanalyst analysis of enrichment pathways in the brains of newborn pups

	Total Cmpd	Hits	Raw p	FDR
Purine Metabolism	74	11	0.00017305	0.010866
Arginine and Proline Metabolism	53	13	0.00024146	0.010866
Aspartate Metabolism	35	9	0.0017542	0.045637
Citric Acid Cycle	32	4	0.0021556	0.045637
Urea Cycle	29	9	0.0033856	0.045637
Carnitine Synthesis	22	6	0.0035261	0.045637
D-Arginine and D-Ornithine Metabolism	11	1	0.0037003	0.045637
Malate-Aspartate Shuttle	10	2	0.0042442	0.045637
Thyroid hormone synthesis	13	1	0.005087	0.045637
Mitochondrial Electron Transport Chain	19	3	0.00554	0.045637
Lysine Degradation	30	1	0.0055778	0.045637
Valine, Leucine and Isoleucine Degradation	60	6	0.0068409	0.051307
Warburg Effect	58	9	0.010471	0.063719
Beta-Alanine Metabolism	34	3	0.010671	0.063719
Amino Sugar Metabolism	33	5	0.011008	0.063719
Starch and Sucrose Metabolism	31	5	0.011328	0.063719
Ammonia Recycling	32	7	0.016075	0.083697
Nicotinate and Nicotinamide Metabolism	37	9	0.017947	0.083697
Fatty Acid Elongation In Mitochondria	35	1	0.018619	0.083697
Plasmalogen Synthesis	26	4	0.018935	0.083697
Phosphatidylcholine Biosynthesis	14	7	0.019529	0.083697
Threonine and 2-Oxobutanoate Degradation	20	1	0.021805	0.087166
Pyruvate Metabolism	48	5	0.023119	0.087166
Pyrimidine Metabolism	59	9	0.023244	0.087166
Fructose and Mannose Degradation	32	4	0.024576	0.088473
Arachidonic Acid Metabolism	69	3	0.028581	0.094649
Phenylalanine and Tyrosine Metabolism	28	6	0.028784	0.094649
Betaine Metabolism	21	7	0.030446	0.094649
Phospholipid Biosynthesis	29	3	0.030498	0.094649
Tryptophan Metabolism	60	7	0.033948	0.10184
Bile Acid Biosynthesis	65	6	0.035603	0.10329
Ubiquinone Biosynthesis	20	3	0.036726	0.10329
Galactose Metabolism	38	10	0.042175	0.10767
Gluconeogenesis	35	6	0.042861	0.10767
Methylhistidine Metabolism	4	2	0.044104	0.10767
Estrone Metabolism	24	2	0.044104	0.10767
Pyruvaldehyde Degradation	10	1	0.044263	0.10767
Phosphatidylethanolamine Biosynthesis	12	5	0.049989	0.11839
Phenylacetate Metabolism	9	3	0.053442	0.12333
Histidine Metabolism	43	8	0.06765	0.14638
Glycerolipid Metabolism	25	5	0.068329	0.14638
Glycolysis	25	4	0.073444	0.14638
Phosphatidylinositol Phosphate Metabolism	17	2	0.074022	0.14638
Steroid Biosynthesis	48	5	0.075285	0.14638
Cardiolipin Biosynthesis	11	3	0.076353	0.14638
Inositol Phosphate Metabolism	26	1	0.078069	0.14638

Trehalose Degradation	11	1	0.078069	0.14638
De Novo Triacylglycerol Biosynthesis	9	1	0.078069	0.14638
Spermidine and Spermine Biosynthesis	18	5	0.087883	0.16142
Lactose Synthesis	20	5	0.097734	0.17592
Alpha Linolenic Acid and Linoleic Acid Metabolis	19	1	0.10035	0.17709
Porphyrin Metabolism	40	2	0.12847	0.22235
Inositol Metabolism	33	2	0.14025	0.23375
Sulfate/Sulfite Metabolism	22	2	0.14025	0.23375
Phytanic Acid Peroxisomal Oxidation	26	2	0.14511	0.23745
Ketone Body Metabolism	13	1	0.15276	0.24551
Biotin Metabolism	8	1	0.17835	0.2816
Mitochondrial Beta-Oxidation of Medium Chain	27	3	0.19988	0.31016
Alanine Metabolism	17	6	0.2155	0.32873
Fatty Acid Biosynthesis	35	5	0.25504	0.38256
Tyrosine Metabolism	72	6	0.27778	0.40984
Nucleotide Sugars Metabolism	20	3	0.29123	0.42275
Sphingolipid Metabolism	40	4	0.31237	0.43501
Transfer of Acetyl Groups into Mitochondria	22	3	0.3153	0.43501
Catecholamine Biosynthesis	20	4	0.31708	0.43501
Fatty acid Metabolism	43	4	0.31901	0.43501
Beta Oxidation of Very Long Chain Fatty Acids	17	4	0.32742	0.43982
Lactose Degradation	9	3	0.36833	0.48749
Propanoate Metabolism	42	4	0.37399	0.48782
Mitochondrial Beta-Oxidation of Long Chain Sat	28	4	0.38578	0.496
Butyrate Metabolism	19	3	0.41652	0.52076
Riboflavin Metabolism	20	3	0.41661	0.52076
Mitochondrial Beta-Oxidation of Short Chain Sa	27	3	0.45525	0.56126
Ethanol Degradation	19	2	0.47459	0.57721
Glycine and Serine Metabolism	59	16	0.48242	0.5789
Folate Metabolism	29	3	0.54173	0.6284
Oxidation of Branched Chain Fatty Acids	26	5	0.5441	0.6284
Glutamate Metabolism	49	11	0.56274	0.6284
Steroidogenesis	43	1	0.56527	0.6284
Pentose Phosphate Pathway	29	2	0.56678	0.6284
Thiamine Metabolism	9	2	0.56678	0.6284
Pterine Biosynthesis	29	1	0.57254	0.6284
Methionine Metabolism	43	12	0.63032	0.68348
Glutathione Metabolism	21	6	0.69142	0.74081
Selenoamino Acid Metabolism	28	3	0.70473	0.74618
Glucose-Alanine Cycle	13	3	0.73977	0.77418
Taurine and Hypotaurine Metabolism	12	2	0.78132	0.80826
Pantothenate and CoA Biosynthesis	21	5	0.79057	0.80853
Homocysteine Degradation	9	2	0.81306	0.82219
Cysteine Metabolism	26	4	0.88194	0.88194

Supplementary Table 3. Adult metabolite differential expressions

Label	pVal	Fold_Change
PE (38:4); PE(19:2/19:2);	0.0012708	-1.323989757
PE (p-38:4); PE(P-16:0/22	0.0033697	-1.93443963
urea	0.005304	-1.360062716
UDP-N-acetylglucosamine	0.0080877	-1.397084048
PE (38:4); PE(16:0/22:4);	0.0099433	-1.46234864
PC (38:4)	0.0110451	-1.418304177
sucrose	0.0115212	1.30734767
PC (o-32:0)	0.0159487	-1.594770029
SM (d36:2)	0.0170569	-1.916587385
PC (40:5)	0.017285	-1.255304313
N-Octadecanoyl-1-.beta.-[illegible]	0.0186709	-1.848020061
SM (d36:1)	0.0204108	-1.782202133
guanosine	0.0252246	1.334669339
PE (38:4)	0.0252532	-1.512904694
PI (36:4)	0.0273519	2.007767354
TG (52:1)	0.0354902	-1.248174839
lyxitol	0.0359114	1.283474853
PE (p-36:4) or PE (o-36:5)	0.0378509	-1.865264077
PC (p-36:3) or PC (o-36:4)	0.0392595	-1.332110441
isoleucine	0.0398941	1.341933802
PC (40:5)	0.0441218	-1.176933406
2-hydroxyglutaric acid	0.044591	-1.877374169
PE (38:2)	0.0465127	-1.234653502
SM (d36:1)	0.046723	-1.478118762
1-Methyladenosine	0.0489158	-1.233539775

Supplementary Table 4. Metaboanalyst analysis of enrichment pathways in the brains of adult pups

	Total Cmpc	Hits	Statistic Q	Raw p	FDR
Oxidation of Branched Chain Fatty Acids	26	4	18.585	0.046582	0.52975
Catecholamine Biosynthesis	20	3	21.657	0.054041	0.52975
Carnitine Synthesis	22	4	13.32	0.11591	0.52975
Phytanic Acid Peroxisomal Oxidation	26	3	14.028	0.11613	0.52975
Tyrosine Metabolism	72	8	11.214	0.15681	0.52975
Glycerolipid Metabolism	25	6	10.638	0.18808	0.52975
D-Arginine and D-Ornithine Metabolism	11	2	11.505	0.19504	0.52975
Thyroid hormone synthesis	13	2	11.367	0.19733	0.52975
Vitamin B6 Metabolism	20	1	11.549	0.19781	0.52975
Vitamin K Metabolism	14	1	11.549	0.19781	0.52975
Beta Oxidation of Very Long Chain Fatty A	17	2	11.269	0.20179	0.52975
Pyruvaldehyde Degradation	10	2	11.003	0.20503	0.52975
Plasmalogen Synthesis	26	5	9.9735	0.20952	0.52975
Citric Acid Cycle	32	5	9.8712	0.22459	0.52975
Caffeine Metabolism	24	2	9.9624	0.22549	0.52975
Glycerol Phosphate Shuttle	11	2	9.9624	0.22549	0.52975
Threonine and 2-Oxobutanoate Degradati	20	3	9.8202	0.22898	0.52975
Mitochondrial Electron Transport Chain	19	4	9.7732	0.23042	0.52975
Alpha Linolenic Acid and Linoleic Acid Met	19	1	10.026	0.23215	0.52975
Porphyrin Metabolism	40	3	9.6479	0.23336	0.52975
Galactose Metabolism	38	7	9.2093	0.23638	0.52975
Pyrimidine Metabolism	59	8	9.0564	0.23979	0.52975
Folate Metabolism	29	4	9.4127	0.24059	0.52975
Lysine Degradation	30	3	9.4143	0.24077	0.52975
Glutathione Metabolism	21	8	8.7365	0.2499	0.52975
Betaine Metabolism	21	9	8.823	0.25124	0.52975
Warburg Effect	58	8	8.8125	0.25666	0.52975
Phospholipid Biosynthesis	29	6	8.6662	0.25774	0.52975
Tryptophan Metabolism	60	6	8.7947	0.25807	0.52975
Lactose Degradation	9	1	8.8011	0.26453	0.52975
Trehalose Degradation	11	1	8.8011	0.26453	0.52975
Transfer of Acetyl Groups into Mitochondi	22	3	8.5611	0.26856	0.52975
Glycolysis	25	2	8.2028	0.28195	0.52975
De Novo Triacylglycerol Biosynthesis	9	2	8.2028	0.28195	0.52975
Pterine Biosynthesis	29	1	8.1994	0.28229	0.52975
Androgen and Estrogen Metabolism	33	1	8.1994	0.28229	0.52975
Ketone Body Metabolism	13	1	8.1994	0.28229	0.52975
Retinol Metabolism	37	1	8.1994	0.28229	0.52975
Androstenedione Metabolism	24	1	8.1994	0.28229	0.52975
Estrone Metabolism	24	1	8.1994	0.28229	0.52975
Starch and Sucrose Metabolism	31	3	8.0357	0.2855	0.52975
Cardiolipin Biosynthesis	11	3	8.0357	0.2855	0.52975
Steroid Biosynthesis	48	6	8.1944	0.28565	0.52975
Nucleotide Sugars Metabolism	20	2	8.0316	0.28582	0.52975
Fructose and Mannose Degradation	32	4	7.9475	0.28646	0.52975
Steroidogenesis	43	2	8.0334	0.2866	0.52975

Fatty Acid Elongation In Mitochondria	35	2	8.0024	0.28739	0.52975
Inositol Phosphate Metabolism	26	3	7.9106	0.28937	0.52975
Inositol Metabolism	33	4	7.7643	0.29316	0.52975
Mitochondrial Beta-Oxidation of Long Cha	28	6	8.0375	0.29415	0.52975
Mitochondrial Beta-Oxidation of Short Cha	27	5	7.9859	0.29736	0.52975
Beta-Alanine Metabolism	34	7	7.898	0.29851	0.52975
Fatty acid Metabolism	43	7	7.852	0.30346	0.52975
Gluconeogenesis	35	4	7.4528	0.30473	0.52975
Valine, Leucine and Isoleucine Degradatio	60	7	7.8596	0.3093	0.52975
Butyrate Metabolism	19	4	7.5989	0.3235	0.52975
Mitochondrial Beta-Oxidation of Medium	27	4	7.5989	0.3235	0.52975
Nicotinate and Nicotinamide Metabolism	37	9	7.612	0.32806	0.52975
Pyruvate Metabolism	48	7	7.4452	0.33103	0.52975
Glucose-Alanine Cycle	13	3	6.8147	0.33414	0.52975
Propanoate Metabolism	42	7	7.2838	0.33971	0.52975
Histidine Metabolism	43	10	7.1888	0.34529	0.52975
Riboflavin Metabolism	20	5	7.2281	0.35467	0.52975
Methionine Metabolism	43	13	7.0257	0.35604	0.52975
Bile Acid Biosynthesis	65	7	6.9964	0.35932	0.52975
Phenylalanine and Tyrosine Metabolism	28	7	7.0395	0.3642	0.52975
Glutamate Metabolism	49	16	6.938	0.37494	0.53513
Ammonia Recycling	32	11	6.4543	0.39611	0.53513
Glycine and Serine Metabolism	59	15	6.54	0.39669	0.53513
Malate-Aspartate Shuttle	10	4	5.6813	0.39678	0.53513
Ethanol Degradation	19	3	5.7819	0.39781	0.53513
Arginine and Proline Metabolism	53	13	6.4106	0.40134	0.53513
Cysteine Metabolism	26	6	5.6173	0.41402	0.53905
Selenoamino Acid Metabolism	28	6	5.6296	0.4214	0.53905
Sulfate/Sulfite Metabolism	22	2	4.8353	0.42675	0.53905
Lactose Synthesis	20	2	4.8353	0.42675	0.53905
Ubiquinone Biosynthesis	20	1	4.3376	0.43891	0.54019
Biotin Metabolism	8	1	4.3376	0.43891	0.54019
Purine Metabolism	74	17	6.2845	0.44906	0.54418
Pentose Phosphate Pathway	29	2	4.0852	0.45348	0.54418
Aspartate Metabolism	35	11	5.6292	0.48041	0.56938
Pantothenate and CoA Biosynthesis	21	4	3.8126	0.49223	0.57627
Phenylacetate Metabolism	9	3	3.7586	0.5061	0.58274
Thiamine Metabolism	9	3	3.7142	0.5099	0.58274
Phosphatidylinositol Phosphate Metabolis	17	2	3.3939	0.52327	0.59099
Urea Cycle	29	11	4.5972	0.5489	0.61273
Taurine and Hypotaurine Metabolism	12	2	2.7326	0.5577	0.61488
Alanine Metabolism	17	6	3.539	0.56364	0.61488
Arachidonic Acid Metabolism	69	3	3.8577	0.62181	0.67072
Spermidine and Spermine Biosynthesis	18	5	3.6026	0.63005	0.67206
Homocysteine Degradation	9	2	3.0202	0.66047	0.69676
Sphingolipid Metabolism	40	3	2.6919	0.70632	0.73703
Fatty Acid Biosynthesis	35	1	0.40413	0.81507	0.84136

Phosphatidylethanolamine Biosynthesis	12	5	2.6725	0.83729	0.85312
Phosphatidylcholine Biosynthesis	14	4	2.1948	0.84423	0.85312
Amino Sugar Metabolism	33	4	1.3816	0.90686	0.90686

Supplementary Table 5. Newborn RBPs and TFs

Gene Symb	Fold	P-val
Cirbp	-1.25	0.0007
A1cf	-1.25	0.0013
Elavl2	1.17	0.0077
Hnrnpk	-1.21	0.0194
Khdrbs3	1.17	0.0232
Pabpc5	1.1	0.0258
Fus	-1.2	0.0259
Cpeb3	1.14	0.0266
Igf2bp3	-1.08	0.0445

Supplementary Table 6. Metaboanalyst analysis of associated diseases with metabolites set

	Total Cmpd	Hits	Raw p	FDR
Rett Syndrome	5	2	0.000101	0.005769
Pyridoxamine 5-Prime-Phosphate	2	2	0.000348	0.008649
Pyruvate Dehydrogenase Deficien	2	1	0.000455	0.008649
Alzheimer's Diesease	7	5	0.001064	0.010933
Bacterial Meningitis	9	4	0.0015	0.010933
Leukemia	21	13	0.001638	0.010933
Degenerative Disc Disease	6	2	0.001816	0.010933
Schizophrenia	24	13	0.002217	0.010933
Hydrocephalus	5	1	0.002224	0.010933
Lesch-Nyhan Syndrome	4	1	0.002224	0.010933
Different Seizure Disorders	25	13	0.002241	0.010933
3-Phosphoglycerate Dehydrogena	2	1	0.002494	0.010933
Juvenile Myoclonic Epilepsy	3	1	0.002494	0.010933
Hypothyroidism	8	3	0.007577	0.030848
Brain Injury	8	4	0.008986	0.034145
Canavan Disease	4	4	0.014479	0.051582
Transurethral Resection Of The Pr	2	1	0.019909	0.066755
Viral Meningitis	5	1	0.024158	0.076502
Epilepsy	14	4	0.02832	0.076615
Traumatic Brain Injury	6	2	0.028851	0.076615
Glut-1 Deficiency Syndrome	2	2	0.034957	0.076615
Aseptic Meningitis	5	4	0.03624	0.076615
Post Transurethral Prostatic Resec	5	3	0.038291	0.076615
Friedreich's Ataxia	5	1	0.038979	0.076615
Hereditary Spastic Paraplegia	5	1	0.038979	0.076615
Olivopontocerebral Atrophy	5	1	0.038979	0.076615
Ataxia (Assorted)	5	1	0.038979	0.076615
Friedreich Ataxia	4	1	0.038979	0.076615
Heredo-Degenerative Ataxias | Sp	5	1	0.038979	0.076615
Hyperphenylalaniemia Due To Gu	5	1	0.046353	0.08807
Acute Tonic-Clonic Seizures	3	2	0.053823	0.098965
Tuberculous Meningitis	7	3	0.064081	0.11414
Alcoholism	5	1	0.06619	0.11433
Parkinson's Disease	14	1	0.11232	0.18346
Meningomyelocoele (Spina Bifida)	8	3	0.1163	0.18346
Meningitis	15	4	0.11673	0.18346
Febrile Seizures	2	2	0.11909	0.18346
Alzheimer's Disease	51	18	0.13167	0.19751
Spinocerebellar Degeneration	3	2	0.15323	0.22395
Gaba-Transaminase Deficiency	2	1	0.19089	0.24729
2-Hydroxyglutaric Aciduria (D)	2	1	0.19089	0.24729
Gaba Transaminase Deficiency	3	1	0.19089	0.24729
Premenstrual Dysphoric Disorder	2	1	0.19089	0.24729
Pyridoxine Dependency With Seizu	3	1	0.19089	0.24729
Complex Febrile Seizures	3	3	0.24101	0.30528
Anoxia	9	3	0.2612	0.32366

Biotinidase Deficiency	6	3	0.26924	0.32653
Propionic Acidemia	7	2	0.30942	0.36744
Early-Onset Encephalopathy And (	2	1	0.40094	0.4664
Multi-Infarct Dementia	6	1	0.43931	0.491
Vascular Dementia	6	1	0.43931	0.491
Aging-Related Metabolites	4	3	0.50945	0.55843
Multiple Sclerosis	13	3	0.74365	0.79977
Dementia	4	2	0.76811	0.81078
Peripheral Neuropathy	2	1	0.91069	0.92696
Stroke	7	1	0.91069	0.92696
Glucose Transporter Type 1 Defici	2	1	0.96323	0.96323

Supplementary Methods

Biogeneic Amines metabolites' Measurement

Summary

The biogenic amine HILIC analysis workflow involves analysis of polar phase of lipid extraction by ultra high pressure liquid chromatography (UHPLC) on a Waters BEH Amide Column, interfaced to a SCIEX Triple TOF 6600 mass spectrometer (high resolution, accurate mass), with a 16.8 minute total run time. Data are collected in both positive and negative ion mode, and analyzed using MS DIAL, open software for metabolome analysis

Sample Preparation

Extraction is carried out using a bi-phasic solvent system of cold methanol, methyl *tert*-butyl ether (MTBE), and water. In more detail, cold methanol (225 µL is added to a 5mg tissue sample aliquot, which is placed into a 1.5 mL Eppendorf tube. Then, 750 µL of cold MTBE is added, followed by vortexing for 10 s. and shaking for 6 min. at 4ºC. Phase separation is induced by adding 188 µL of mass spec-grade water. After vortexing for 20 s. the sample is centrifuged at 14,000 rpm for 2 min. The upper organic phase is collected in two 300 µL aliquots for lipid analysis polar layer is collected in two 125 µL aliquots for HILIC analysis. One is stored at -20ºC as a backup and the other is evaporated to dryness in a SpeedVac. Dried extracts are resuspended in acetonitrile.

LC/MS parameters

The LC/QTOFMS analyses are performed using an Agilent 1290 Infinity LC system (G4220A binary pump, G4226A autosampler, and G1316C Column Thermostat) coupled to a SCIEX Triple TOF mass spectrometer. Polar compounds are separated on an Acquity UPLC BEH Amide Column, 130Å, 1.7 µm, 2.1 mm X 150 mm maintained at 45°C at a flow-rate of 0.4 mL/min. Solvent pre-heating (Agilent G1316) was used. The mobile phases consist of: Water, 10 mM Ammonium Formate, 0.125% Formic Acid (A) and Acetonitrile: Water (95/5, v/v), 10 mM Ammonium Formate, 0.125% Formic Acid (B)

The gradient is as follows: 0 min 100% (A); 0–2 min 100% (A); 2–7.7 min 30% (A); 7.7–9.5 min 60% (A); 9.5–10.3 min 70% (A); 10.3–12.8 min 0% (A); 12.8–16.8 min 0% (A. A sample volume of 1 µL for positive mode and 3 µL for negative mode is used for the injection. Sample temperature is maintained at 4°C in the autosampler.

SCIEX Triple TOF 6600mass spectrometers are operated with electrospray ionization (ESI) performing full scan in the mass range m/z 50–1200. Number of cycles in MS1 is 1667 with cycle time of 500ms and accumulation time 475ms. Mass spectrometer parameters are as follows (positive mode) Gas Temp 300°C, gas pressures in psi units with : GS1 and GS2 50 psi, CUR: 35. ISVF is 4500V and DP and CE are 10V and 100 V.

Supplementary References

1. Matyash, V., et al., *Lipid extraction by methyl-tert-butyl ether for high-throughput lipidomics.* J Lipid Res, 2008. **49**(5): p. 1137-46.
2. Tsugawa, H.,et al., MS-DIAL: data-independent MS/MS deconvolution for comprehensive metabolome analysis. Nature Methods 12, p 523–526 (2015)

Lipidomics Measurement Method

Summary

The lipidomics workflow involves sample extraction in MTBE with addition of internal standards, followed by ultra high pressure liquid chromatography (UHPLC) on a Waters CSH column, interfaced to a QTOF mass spectrometer (high resolution, accurate mass), with a 15 minute total run time. Data are collected in both positive and negative ion mode, and analyzed using MassHunter (Agilent). Approximately 400 lipids can be identified from plasma, with additional unknowns. The method is highly stable and has been validated on large datasets (>8,000 samples) collected over long time periods (> 1 year).

Sample Preparation

Extraction of plasma lipids is based on the "Maytash" method [1] which was subsequently modified. Extraction is carried out using a bi-phasic solvent system of cold methanol, methyl *tert*-butyl ether (MTBE), and water. In more detail, cold methanol (225 µL) containing a mixture of odd chain and deuterated lipid internal standards [lysoPE(17:1), lysoPC(17:0), PC(12:0/13:0), PE(17:0/17:0), PG(17:0/17:0), sphingosine (d17:1), d_7-cholesterol, SM(17:0), C17 ceramide, d_3-palmitic acid, MG(17:0/0:0/0:0), DG(18:1/2:0/0:0), DG(12:0/12:0/0:0), and d_5-TG(17:0/17:1/17:0)] is added to a 20 µL sample aliquot, which is placed into a 1.5 mL Eppendorf tube, and the tube is vortexed for 10 s. Then, 750 µL of cold MTBE containing CE(22:1) (internal standard) are added, followed by vortexing for 10 s. and shaking for 6 min. at 4ºC. Phase separation is induced by adding 188 µL of mass spec-grade water. After vortexing for 20 s. the sample is centrifuged at 14,000 rpm for 2 min. The upper organic phase is collected in two 300 µL aliquots. One is stored at -20ºC as a backup and the other is evaporated to dryness in a SpeedVac. Dried extracts are resuspended using a mixture of methanol/toluene (9:1, v/v) (60 µL) containing an internal standard [12-[[(cyclohexylamino)carbonyl]amino]-dodecanoic acid (CUDA)] used as a quality control.

LC/MS parameters

The LC/QTOFMS analyses are performed using an Agilent 1290 Infinity LC system (G4220A binary pump, G4226A autosampler, and G1316C Column Thermostat) coupled to either an Agilent 6530 (positive ion mode) or an Agilent 6550 mass spectrometer equipped with an ion funnel (iFunnel) (negative ion mode). Lipids are separated on an Acquity UPLC CSH C18 column (100 x 2.1 mm; 1.7 µm) maintained at 65°C at a flow-rate of 0.6 mL/min. Solvent pre-heating (Agilent G1316) was used. The mobile phases consist of 60:40 acetonitrile:water with 10 mM ammonium formate and 0.1% formic acid (A) and 90:10 propan-2-ol:acetonitrile with 10 mM ammonium formate and 0.1% formic acid. The gradient is as follows: 0 min 85% (A); 0–2 min 70% (A); 2–2.5 min 52% (A); 2.5–11 min 18% (A); 11–11.5 min 1% (A); 11.5–12 min 1% (A); 12–12.1 min 85% (A); 12.1–15 min 85% (A). A sample volume of 3 µL is used for the injection. Sample temperature is maintained at 4°C in the autosampler.

The quadrupole/time-of-flight (QTOF) mass spectrometers are operated with electrospray ionization (ESI) performing full scan in the mass range *m*/*z* 65–1700 in positive (Agilent 6530, equipped with a JetStreamSource) and negative (Agilent 6550, equipped with a dual JetStream Source) modes producing both unique and complementary spectra. Instrument paramaters are as follows (positive mode) Gas Temp 325°C, Gas Flow 8 l/min, Nebulizer 35 psig, Sheath Gas 350°C, Sheath Gas Flow 11, Capillary Voltage 3500 V, Nozzle Voltage 1000V, Fragmentor 120V, Skimmer 65V. Data (both profile and centroid) are collected at a rate of 2 scans per second. In negative ion mode, Gas Temp 200°C, Gas Flow 14 l/min, Fragmentor 175V, with the other parameters identical to positive ion mode. For the 6530 QTOF, a reference solution generating ions of 121.050 and 922.007 *m/z* in positive mode and 119.036 and 966.0007 m/z in negative mode, and these are used for continuous mass correction. For the 6550, the reference solution is introduced via a dual spray ESI, with the same ions and continuous mass correction.

Samples are injected (1.7 µl in positive mode and 5 µl in negative ion mode) with a needle wash for 20 seconds (wash solvent is isopropanol). The valve is switched back and forth during the run for washing; this has been shown to be essential for reducing carryover of less polar lipids.

Data Analysis

For the data processing the MassHunter software is used, and a unique ID is given to each lipid based on its retention time and exact mass (RT_mz). This allows the report of peak areas/heights or concentration of lipids based on the use of particular internal standards. Lipids are identified based on their unique MS/MS fragmentation patterns using in-house software, Lipidblast. Using complex lipid class-specific internal standards this approach is used to quantify >400 lipid species including: mono-, di- and triacylglycerols, glycerophospholipids, sphingolipids, cholesterol esters, ceramides, and fatty acids.

This approach is highly reproducible, displaying an RSD of 0.1% for the retention time and 1.7% for peak area based on replicate analysis of plasma samples (*n*=10). An average shot-to-shot carryover of less than 0.1% is observed. The described method is specifically developed to deal with high-throughput analyses, and can be used to analyze over 300 samples a week, including both positive and negative mode data acquisition.

Supplementary References

1. Matyash, V., et al., *Lipid extraction by methyl-tert-butyl ether for high-throughput lipidomics.* J Lipid Res, 2008. **49**(5): p. 1137-46.

Primary metabolites measurement

GC-TOF Method:

Instruments:

Gerstel CIS4 –with dual MPS Injector/

Agilent 6890 GC-

Pegasus III TOF MS

Injector conditions:

Agilent 6890 GC is equipped with a Gerstel automatic liner exchange system (ALEX) that includes a multipurpose sample (MPS2) dual rail, and a Gerstel CIS cold injection system (Gerstel, Muehlheim, Germany) with temperature program as follows: 50°C to 275°C final temperature at a rate of 12 °C/s and hold for 3 minutes. Injection volume is 0.5 μl with 10 μl/s injection speed on a splitless injector with purge time of 25 seconds. Liner (Gerstel #011711-010-00) is changed after every 10 samples, (using the Maestro1 Gerstel software vs. 1.1.4.18). Before and after each injection, the 10 μl injection syringe is washed three times with 10 μl ethyl acetate.

Gas Chromatography conditions:

A 30 m long, 0.25 mm i.d. Rtx-5Sil MS column (0.25 μm 95% dimethyl 5% diphenyl polysiloxane film) with additional 10 m integrated guard column is used (Restek, Bellefonte PA). 99.9999% pure Helium with built-in purifier (Airgas, Radnor PA) is set at constant flow of 1 ml/min. The oven temperature is held constant at 50°C for 1 min and then ramped at 20°C/min to 330°C at which it is held constant for 5 min.

Mass spectrometer settings:

A Leco Pegasus IV time of flight mass spectrometer is controlled by the Leco ChromaTOF software vs. 2.32 (St. Joseph, MI). The transfer line temperature between gas chromatograph and mass spectrometer is set to 280°C. Electron impact ionization at 70V is employed with an ion source temperature of 250°C. Acquisition rate is 17 spectra/second, with a scan mass range of 85-500 Da.
