# Supplementary material for: Metabolomic and transcriptomic signatures of prenatal excessive methionine support nature rather than nurture in schizophrenia pathogenesis
Source: Commun Biol. 2020 Jul 30;3:409. doi: 10.1038/s42003-020-01124-8 (PMC7393105; doi:10.1038/s42003-020-01124-8)
Supplement: Supplementary file 2 — Description of Additional Supplementary Files [file 42003_2020_1124_MOESM2_ESM.pdf]

Descriptions of Additional Supplementary Files

Supplementary Data 1. Gene differential expressions in the brains of newborn SAL vs MET mice

Supplementary Data 2. Top meta pathways, gene-gene enrichment in the brains of the newborn MET pups

Supplementary Data 3. Genes whose expressions were changed in MET brains and occur in metabolites whose levels were changed in MET brains

Supplementary Data 4. Common genes in MET mice and human Schizophrenia and Autism

Supplementary Data 5. Source data underlying the graphs
