# Supplementary material for: Metabolomic and transcriptomic signatures of prenatal excessive methionine support nature rather than nurture in schizophrenia pathogenesis
Source: Commun Biol. 2020 Jul 30;3:409. doi: 10.1038/s42003-020-01124-8 (PMC7393105; doi:10.1038/s42003-020-01124-8)
Supplement: Supplementary file 7 — Supplementary Data 5 [file 42003_2020_1124_MOESM7_ESM.pdf]

Source data underlying the graphs

Supplementary data 5, source data for Figure 1: a, b, c: Maternal behavior of SAL and MET mothers

a. Pups retrieval latency		b. Pups retrieval duration		c. Nest quality score	
SAL	MET	SAL	MET	SAL	MET
44	16	98	51	4	4
17	12	61	36	3.5	4.5
11	17	30	77	4	3
8	32	38	120	4.5	3.5
12	16	62	92	4	3

Supplementary data 5, source data for Figure 1e: Locomotor Activity

SAL→SAL	MET→MET	SAL→MET	MET→SAL
2529.52	3703.6	2720.99	3640.25
3871.88	10808.83	2260.47	5777.77
3340.75	3189.16	1397.75	8087.99
3855.05	7287.93	3589.64	6565.62
5765.09	8126.37	1953.09	5254.19
4519.13	6753.05	4786.89	6551.21
4459.98	9790.09	5747.96	9557.18
	3758.96	2895.25	9188.2
		3804.51	9809.96

Supplementary data 5, source data for Figure 1f: Prepulse inhibition (PPI)

PPI	Group	Mean	Std. Deviation	Std. Error of Mean
68 dB	SAL→SAL	43.47	17.52	5.284
	MET→MET	24.98	20.98	5.82
	SAL→MET	36.72	16.91	3.527
	MET→SAL	16.73	11	2.592
71 dB	SAL→SAL	53.09	24.5	7.386
	MET→MET	30.79	29.63	8.219
	SAL→MET	51.26	17.85	3.644
	MET→SAL	29.52	15.81	3.727
77 dB	SAL→SAL	72.81	11.7	3.701
	MET→MET	51.7	18.98	5.479
	SAL→MET	68.98	15.91	3.247
	MET→SAL	50.91	17.5	4.243

Supplementary data 5, source data for Figure 1g, T-maze: Alternation %

SAL→SAL	MET→MET	SAL→MET	MET→SAL
57.1	57.1	42.9	71.4
85.7	57.1	71.4	28.6
85.7	14.3	71.4	14.3
57.1	57.1	42.9	57.1
71.4	57.1	57.1	57.1
85.7	42.9	71.4	85.7
57.1	57.1	57.1	57.1
71.4	57.1	57.1	14.3
57.1	71.4	42.9	14.3
71.4	28.6	100	14.3
71.4	28.6	85.7	42.9
71.4	57.1	71.4	14.3
71.4	42.9	42.9	28.6
85.7	42.9	42.9	

Supplementary data 5, source data for Figure 1h, T-maze: Decision latency

SAL→SAL	MET→MET	SAL→MET	MET→SAL
7.5	9.5	6.8	7.1
9.1	7.6	5.5	7.6
10.4	9.4	12.8	30.3
7.8	8.9	9.9	6.1
16.6	10.4	6.9	11.1
7.5	10	5.8	5.8
8.9	9	6.8	16.9
12.8	9.3	7.3	11
5.6	15.1	6.3	13.1
5.8	5.8	7.6	8.5
7.3	8.9	10.5	4.5
14.1	10.9	24	6.5
5.3	8.5	8.5	9.8
6.8	13.9	6.6	
