# Supplementary material for: Metabolomic and transcriptomic signatures of prenatal excessive methionine support nature rather than nurture in schizophrenia pathogenesis
Source: Commun Biol. 2020 Jul 30;3:409. doi: 10.1038/s42003-020-01124-8 (PMC7393105; doi:10.1038/s42003-020-01124-8)
Supplement: Supplementary file 8 — Reporting Summary [file 42003_2020_1124_MOESM8_ESM.pdf]

nature research

Corresponding author(s): Amal Alachkar
Last updated by author(s): May 24, 2020

Reporting Summary

Nature Research wishes to improve the reproducibility of the work that we publish. This form provides structure for consistency and transparency in reporting. For further information on Nature Research policies, see Authors & Referees and the Editorial Policy Checklist.

Statistics

For all statistical analyses, confirm that the following items are present in the figure legend, table legend, main text, or Methods section.

n/a	Confirmed	
☐	☒	The exact sample size (*n*) for each experimental group/condition, given as a discrete number and unit of measurement
☐	☒	A statement on whether measurements were taken from distinct samples or whether the same sample was measured repeatedly
☐	☒	The statistical test(s) used AND whether they are one- or two-sided<br>*Only common tests should be described solely by name; describe more complex techniques in the Methods section.*
☒	☐	A description of all covariates tested
☐	☒	A description of any assumptions or corrections, such as tests of normality and adjustment for multiple comparisons
☐	☒	A full description of the statistical parameters including central tendency (e.g. means) or other basic estimates (e.g. regression coefficient) AND variation (e.g. standard deviation) or associated estimates of uncertainty (e.g. confidence intervals)
☐	☒	For null hypothesis testing, the test statistic (e.g. *F*, *t*, *r*) with confidence intervals, effect sizes, degrees of freedom and *P* value noted<br>*Give P values as exact values whenever suitable.*
☒	☐	For Bayesian analysis, information on the choice of priors and Markov chain Monte Carlo settings
☒	☐	For hierarchical and complex designs, identification of the appropriate level for tests and full reporting of outcomes
☒	☐	Estimates of effect sizes (e.g. Cohen's *d*, Pearson's *r*), indicating how they were calculated

Our web collection on statistics for biologists contains articles on many of the points above.

Software and code

Policy information about availability of computer code

Data collection	NA
Data analysis	Transcriptome Analysis Console (TAC) software (Affymetrix)<br>Cyber-T program

For manuscripts utilizing custom algorithms or software that are central to the research but not yet described in published literature, software must be made available to editors/reviewers. We strongly encourage code deposition in a community repository (e.g. GitHub). See the Nature Research guidelines for submitting code & software for further information.

Data

Policy information about availability of data

All manuscripts must include a data availability statement. This statement should provide the following information, where applicable:

- Accession codes, unique identifiers, or web links for publicly available datasets
- A list of figures that have associated raw data
- A description of any restrictions on data availability

The raw data of transcriptomic and metabolomic studies are available in the supplemental materials. Other datasets generated during the current study can be requested from the corresponding author.

Field-specific reporting

Please select the one below that is the best fit for your research. If you are not sure, read the appropriate sections before making your selection.

☒ Life sciences ☐ Behavioural & social sciences ☐ Ecological, evolutionary & environmental sciences

For a reference copy of the document with all sections, see nature.com/documents/nr-reporting-summary-flat.pdf

Life sciences study design

All studies must disclose on these points even when the disclosure is negative.

Sample size	Sample sizes for behavioral and transcriptmic analyses were based on our previous studies on methionine mouse model
Data exclusions	No data were excluded
Replication	Behavioral experiments were carried out using different batches of animals to ensure the reproducibility of the results.
Randomization	SAL and MET offspring used for behavioral, metabolomic, and transcriptomic analyses were randomly selected from a pool of offspring of five MET and five SAL treated mothers.
Blinding	Behavioral tests that required scoring by humans, were analyzed by two individuals who were blind to the group treatment (SAL/MET)

Reporting for specific materials, systems and methods

We require information from authors about some types of materials, experimental systems and methods used in many studies. Here, indicate whether each material, system or method listed is relevant to your study. If you are not sure if a list item applies to your research, read the appropriate section before selecting a response.

Materials & experimental systems

n/a	Involved in the study	
☐	☒	Antibodies
☒	☐	Eukaryotic cell lines
☒	☐	Palaeontology
☐	☒	Animals and other organisms
☒	☐	Human research participants
☒	☐	Clinical data

Methods

n/a	Involved in the study	
☒	☐	ChIP-seq
☒	☐	Flow cytometry
☒	☐	MRI-based neuroimaging

Antibodies

Antibodies used	Chicken anti GFAP (Aves)<br>Rabbit anti KCNMA1 (Sigma-Aldrich)<br>Rabbit anti Arc (abcam)<br>Goat anti rabbit AlexaFluor488 (Invitrogen)<br>Goat anti rabbit AlexaFluor555 (Invitrogen)
Validation	GFAP: Aves cited over 23 studies that used the chicken anti GFAP antibody<br>Rabbit anti KCNMA1: We selected this antibody based on its citation by several papers<br>Arc antibody: we selected this antibody based on its citation by several papers

Animals and other organisms

Policy information about studies involving animals; ARRIVE guidelines recommended for reporting animal research

Laboratory animals	Swiss Webster mice
Wild animals	NA
Field-collected samples	NA
Ethics oversight	The study was approved by the institutional animal care and use committee IACUC at the University of California Irvine

Note that full information on the approval of the study protocol must also be provided in the manuscript.
